# Supplementary material for: Vasoactive Intestinal Peptide Receptor, CRTH2, Antagonist Treatment Improves Eosinophil and Mast Cell-Mediated Esophageal Remodeling and Motility Dysfunction in Eosinophilic Esophagitis
Source: Cells. 2024 Feb 6;13(4):295. doi: 10.3390/cells13040295 (PMC10886969; doi:10.3390/cells13040295)
Supplement: Supplementary file 1 [file cells-13-00295-s001.zip › supplementary figure.pdf]

Supplementary Figure S1

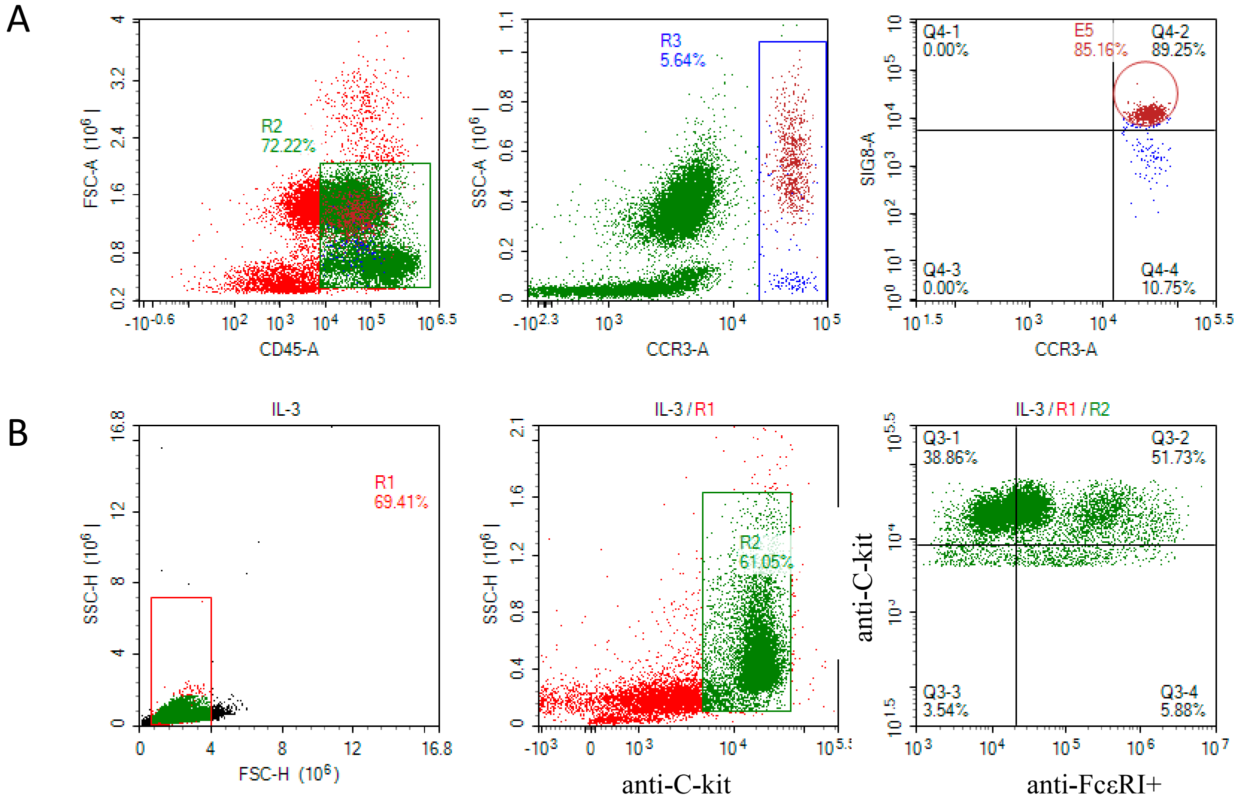

Suppl. Figure S1. Flow study of human blood eosinophils using anti-CCR3 and anti-Siglec8 antibodies. The Siglec8+/CCR3+ double positive human blood eosinophil histogram (A). Mast cells were detected using anti-Ckit and anti-FcεRI+ antibodies in a flow cytometer examination of in vitro mast cells. A histogram of mast cells with anti-cKit+/anti-FcεRI+ double positivity is shown B).

Supplementary Figure S2

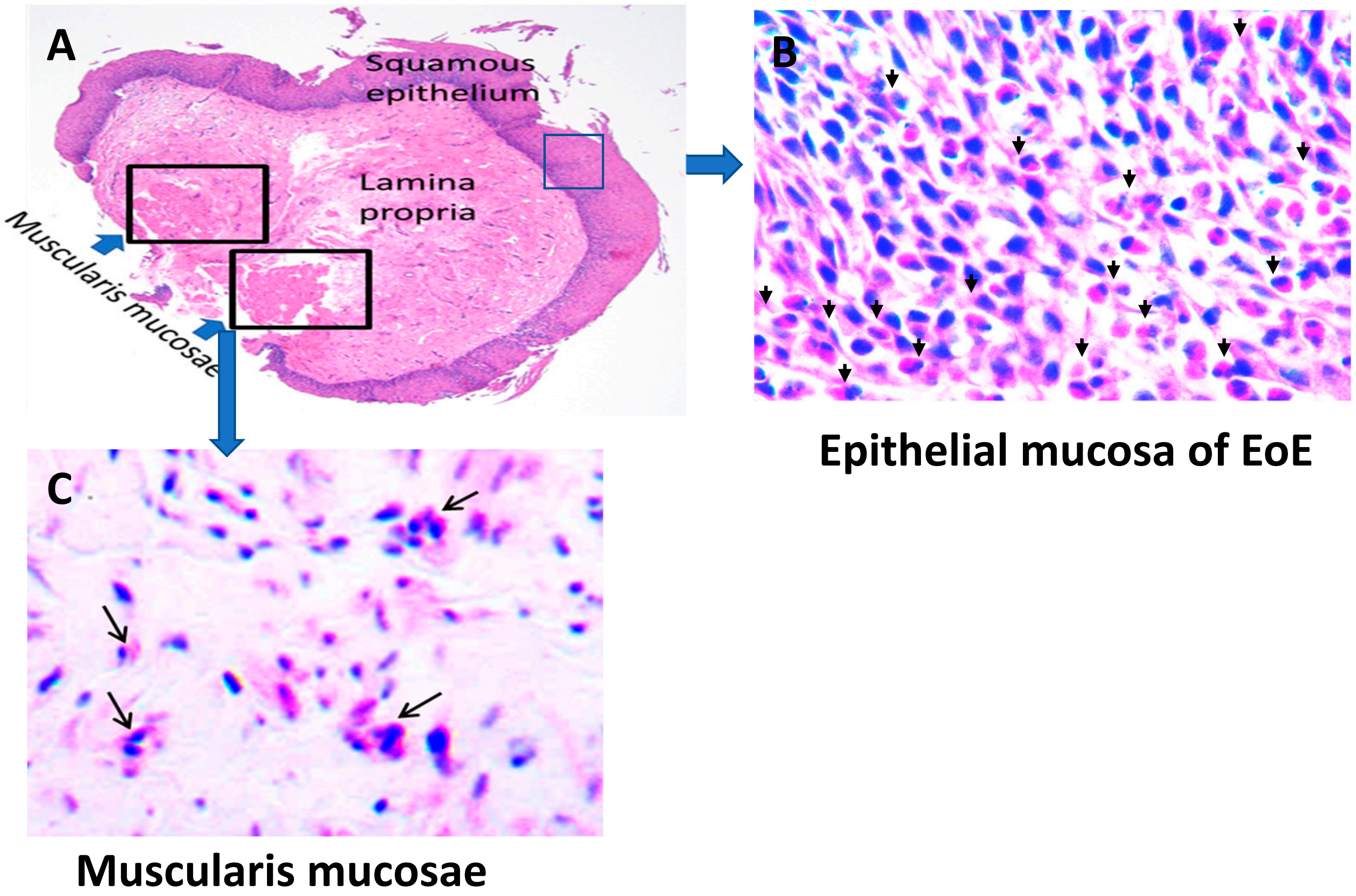

Supplementary Figure S2. Eosinophils accumulate adjacent to the nerve cells in the muscular mucosa of human gastrointestinal disorder. Eosinophil accumulation was detected nearby the nerve cells in the muscularis mucosa of esophageal autopsy section following hematoxylin and eosin staining (a) and muscularis propria of colon biopsies indicated by arrows (b, c). Nerve cells and ganglion are respectively marked with arrowheads and asterisk in the myenteric plexus in colon biopsies (b, c).

Supplementary Figure S3

Experimental EoE model

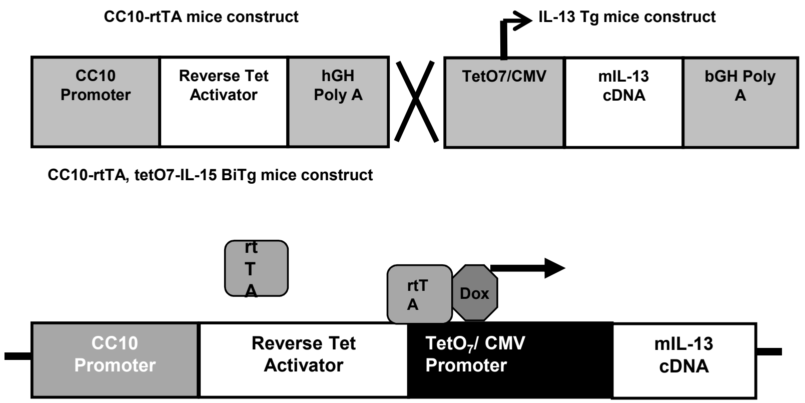

Suppl. Figure S3. Constructs utilized in the generation of CC10-rtTA-IL-13 mice.

Supplementary Figure S4

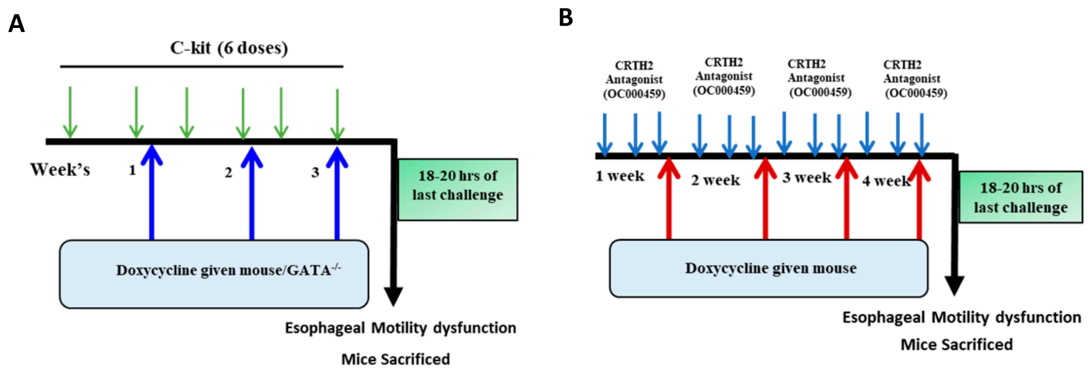

Suppl. Figure S4. Experimental design for checking motility dysfunction.
