# Supplementary material for: Vasoactive Intestinal Peptide Receptor, CRTH2, Antagonist Treatment Improves Eosinophil and Mast Cell-Mediated Esophageal Remodeling and Motility Dysfunction in Eosinophilic Esophagitis
Source: Cells. 2024 Feb 6;13(4):295. doi: 10.3390/cells13040295 (PMC10886969; doi:10.3390/cells13040295)
Supplement: Supplementary file 1 [file cells-13-00295-s001.zip › Supplementary Tables.pdf]

Supplementary Table S1. Patients Clinical and pathological characteristics.

Patients	Age	Gende r	Esophageal Disease	Allergic Diseases	Other Diseases	Eos/HPF	Current Treatment	Steroids
1	9	M	NL	None	None	0		-
2	11	F	NL	None	None	0		-
3	12	F	NL	None	None	0		-
4	9	F	NL	None	None	0		-
5	4	M	NL	None	None	0		-
6	13	M	NL	None	None	0	Elimination diet	Nasocort, Flovent
7	10	F	NL	None	Celiac Disease	0	Elimination diet	-
8	11	M	NL	None	None	0	Food Trial	-
9	12	M	EoE	Eosinophilic gastroenteritis	None	>40/HPF	Elemental + Food	-
10	2	M	EoE	None	None	35 eos/HPF	Elimination	-
11	9	M	EoE	None	None	40 eos/HPF	Elimination	-
12	8	M	EoE	None	None	31 eos/HPF	Ad Lib	-
13	10	M	EoE	None	None	41 eos/HPF	Ad Lib	Flovent
14	12	M	EoE	None	None	63 eos/HPF	Elimination	Nasocort
15			EoE	None	None	30 eos/HPF	-	Flovent
16	3	M	EoE	None	None	30 Eos/ HPF	Ad Lib	Pulmicort
17	11	F	EoE	Eosinophilic gastroenteritis	None	33ceos/HPF	Ad Lib	Beclovent
18	4	F	EoE	Eosinophilic gastroenteritis	None	40 eos/HPF	Elimination	-
19	5	M	EoE with dysphagia	None	None	63 eos/HPF	-	Flovent
20	10	F	EoE with dysphagia	None	None	80 eos/HPF	Ad Lib	-
21	6	M	EoE with dysphagia	None	None	64 eos/HPF	-	Rhinocort, Flovent
22	7	M	EoE with dysphagia	None	non-specific colitis with focal cryptitis	73 eos/HPF	Elimination	Flonase
23	15	M	EoE with dysphagia	None	None	70 eos/HPF	Elimination	-
24	8	M	EoE with dysphagia	None	None	50 eos/HPF	Ad Lib	-
25	11	M	EoE with dysphagia	Eosinophilic gastroenteritis	None	52 eos/HPF	Elimination	-

Abbreviations: NL=normal, M=male, F= female, eos= eosinophils, HPF=high power field, EoE= eosinophilic esophagitis,

Supplementary Table S2. Primers used to quantitate VIP, VPAC-1, VPAC-2, CRTH2 & GAPDH.

Genes	Sense and Anti-Sense Primer Sequence
hVIP	5’-AACCAGAACAGTCAGCTCCG
	5’-CCCTCACTGCTCCTCTTTCC
hVPAC-1	5’-CCCCCTGCTGGGTCTTCTGC
	5’- ATTCGCTGGTGGCTGCCTTCTCAT
hVPAC-2	5’-CTGCACGGTGCCCTGCCCAAAAGT
	5’-GCCCCTCCACCAGCAGCCAGAAGA
hCRTH2	5-AATCCTGTGCTCCCTCTGTGCCCA
	5-GGGAAGCAGAGGCCAACAGGTCG
GAPDH	5'-ACCCAGAAGACTGTGGATGG
	5'-CACATTGGGGGTAGGAACAC
